# Supplementary material for: Inverse Doppler Effects in Pipe Instruments
Source: Sci Rep. 2018 Dec 13;8:17833. doi: 10.1038/s41598-018-36517-7 (PMC6292856; doi:10.1038/s41598-018-36517-7)
Supplement: Supplementary file 1 — Supplementary information [file 41598_2018_36517_MOESM1_ESM.pdf]

Inverse Doppler Effects in Pipe Instruments

S. L. Zhai[1, 3], J. Zhao [2, 3], F. L. Shen[1], L. L. Li[1] & X. P. Zhao[1]

[1]Smart Materials Laboratory, Department of Applied Physics, Northwestern Polytechnical University, Xi'an 710129 P. R. China. [2] Medtronic plc, Boulder, CO 80301, USA. [3]These authors contributed equally to this work. Correspondence and requests for materials should be addressed to J.Z. (email: zhaojing1120@gmail.com) and X.P.Z. (email: xpzhao@nwpu.edu.cn).

Note 1. Measured whole Doppler shift data of the recorder for different pitches at the moving speed of 0.5 m/s...2

Note 2. Measured whole Doppler shift data of the recorder for different pitches at the moving speed of 0.1 m/s...3

Note 3. Measured whole Doppler shift data of the clarinet for different pitches at the moving speed of 0.5 m/s...5

Note 4. Measured whole Doppler shift data of the clarinet for different pitches at the moving speed of 0.1 m/s...6

Note 1. Measured whole Doppler shift data of the recorder for different pitches at the moving speed of 0.5 m/s

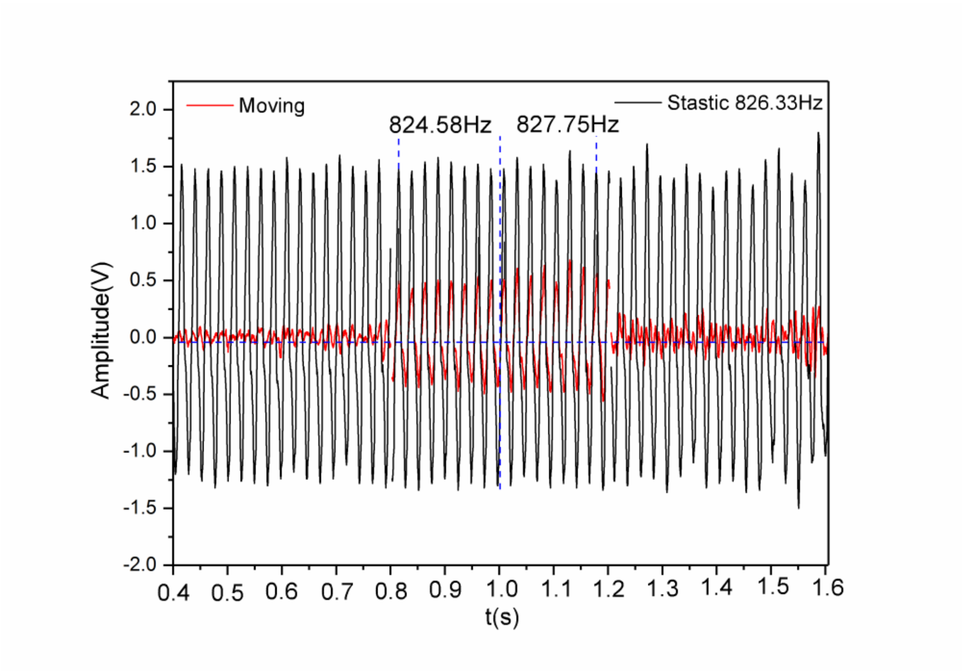

Supplementary Figure S1. The results for pitch 2 at the speed of 0.5 m/s

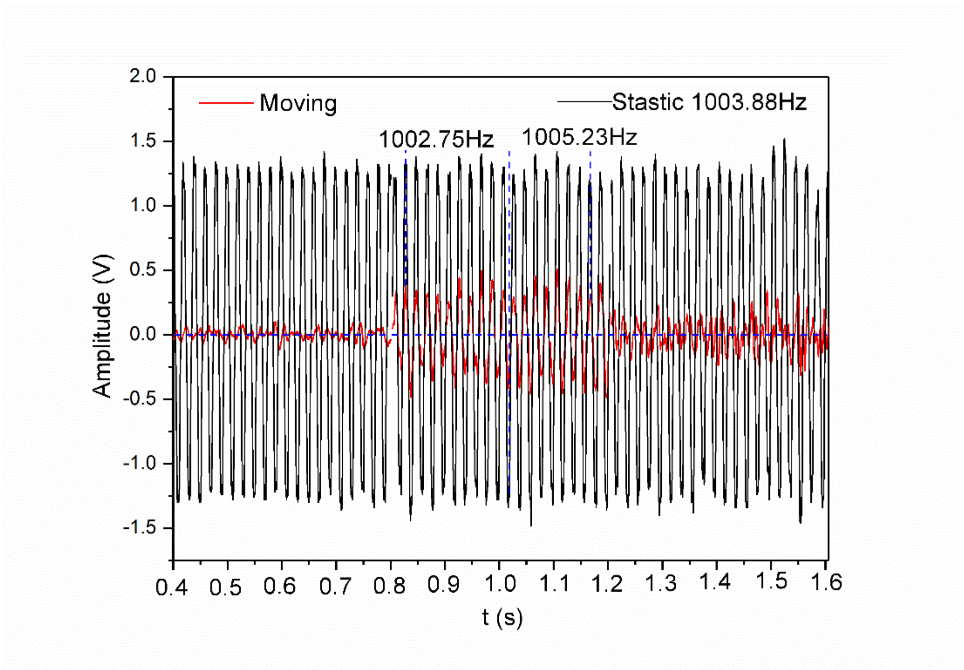

Supplementary Figure S2. The results for pitch 4 at the speed of 0.5 m/s

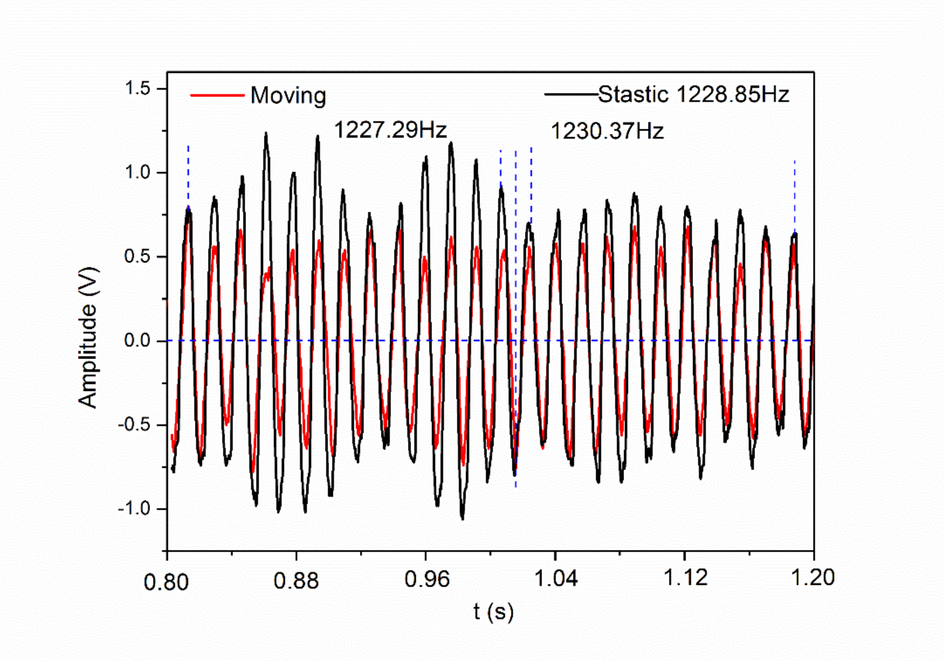

Supplementary Figure S3. The results for pitch 6 at the speed of 0.5 m/s

Note 2. Measured whole Doppler shift data of the recorder for different pitches at the moving speed of 0.1 m/s

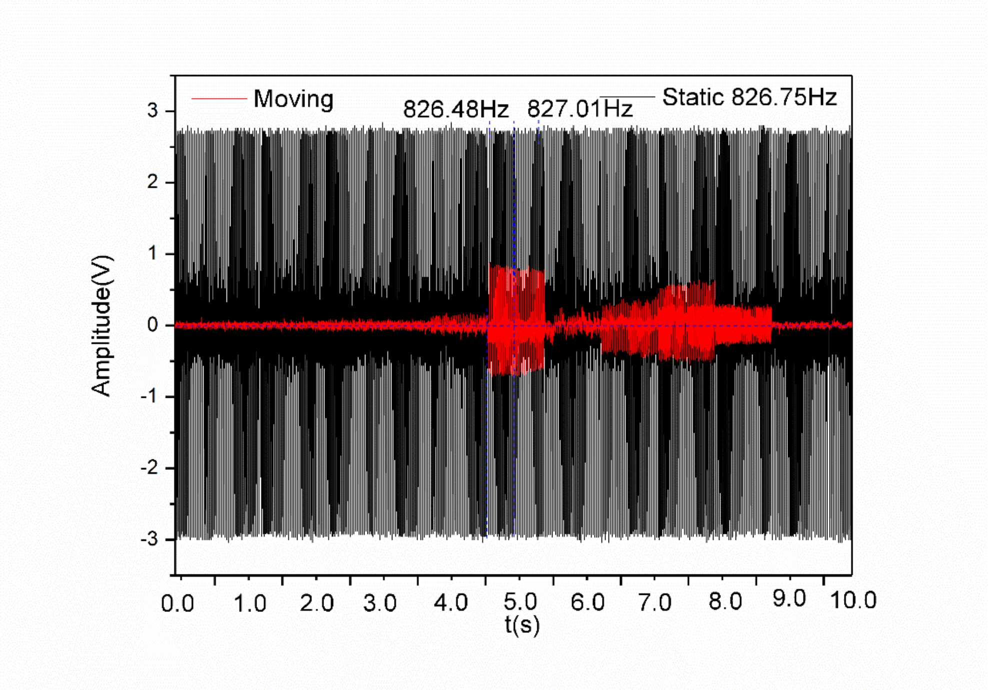

Supplementary Figure S4. The results for pitch 2 at the speed of 0.1 m/s

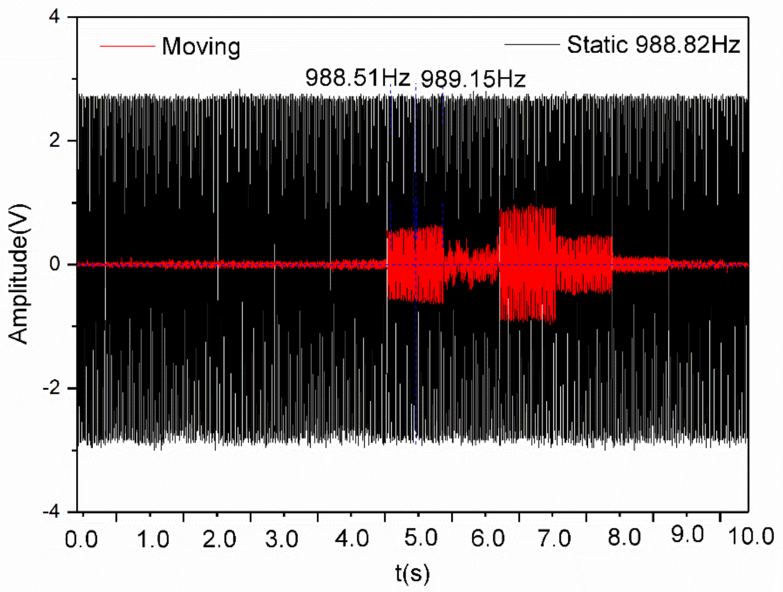

Supplementary Figure S5. The results for pitch 4 at the speed of 0.1 m/s

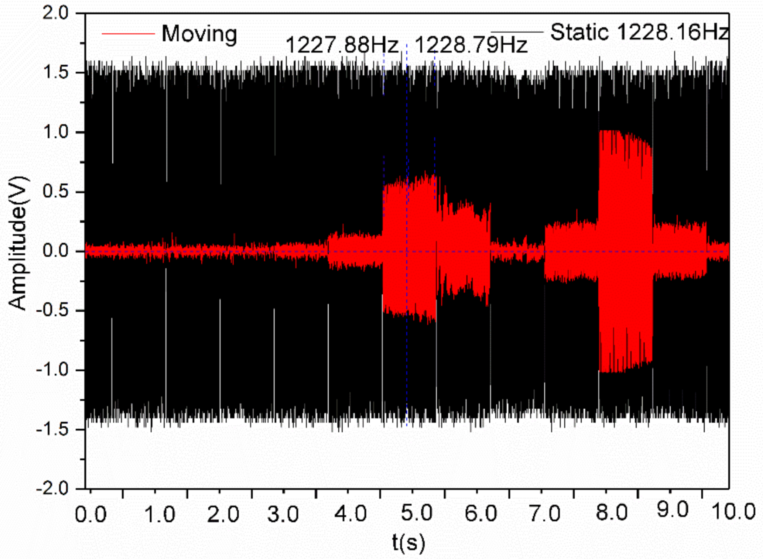

Supplementary Figure S6. The results for pitch 6 at the speed of 0.1 m/s

Note 3. Measured whole Doppler shift data of the clarinet for different pitches at the moving speed of 0.5 m/s

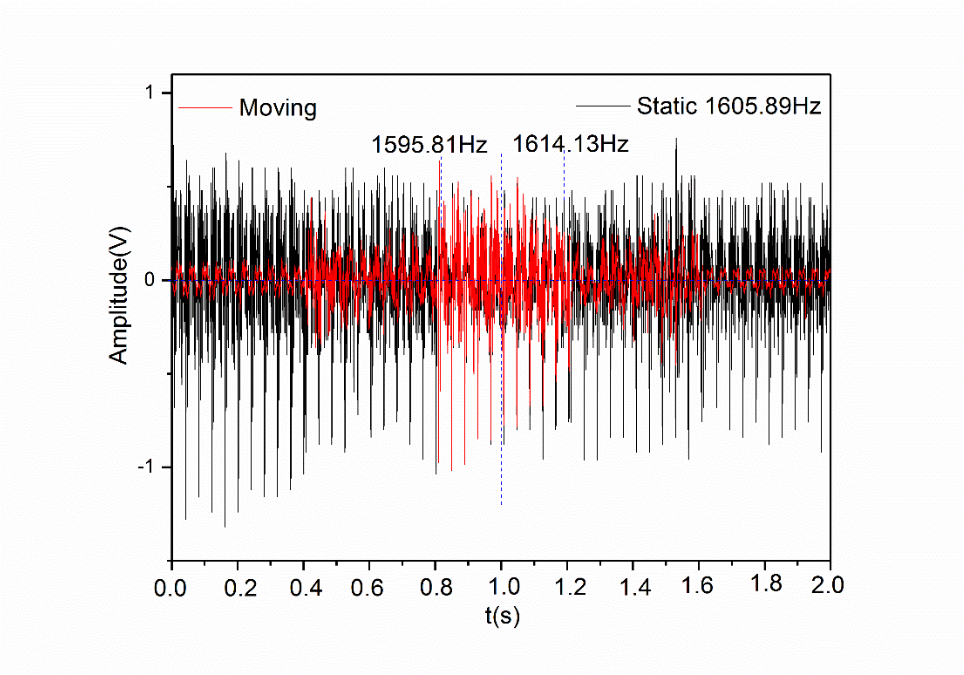

Supplementary Figure S7. The results for pitch 2 at the speed of 0.5 m/s

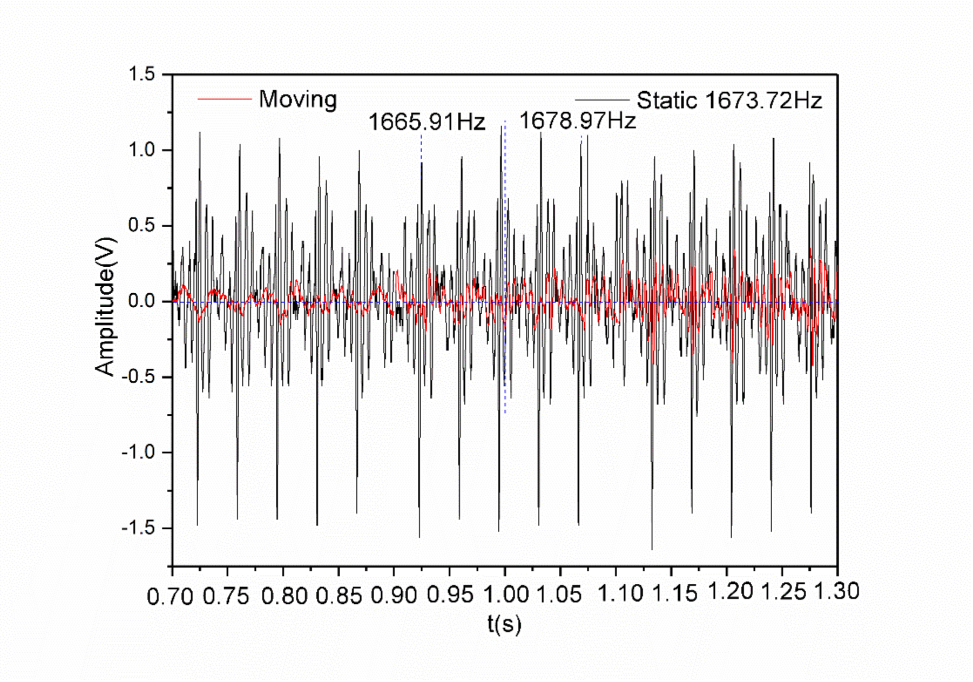

Supplementary Figure S8. The results for pitch 3 at the speed of 0.5 m/s

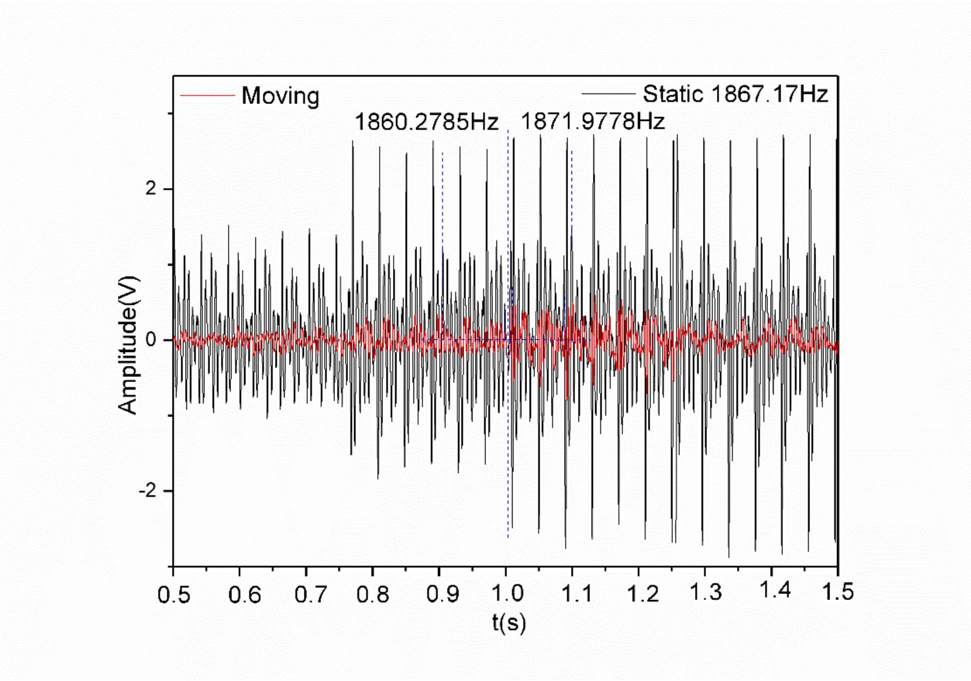

Supplementary Figure S9. The results for pitch 5 at the speed of 0.5 m/s

Note 4. Measured whole Doppler shift data of the clarinet for different pitches at the moving speed of 0.1 m/s

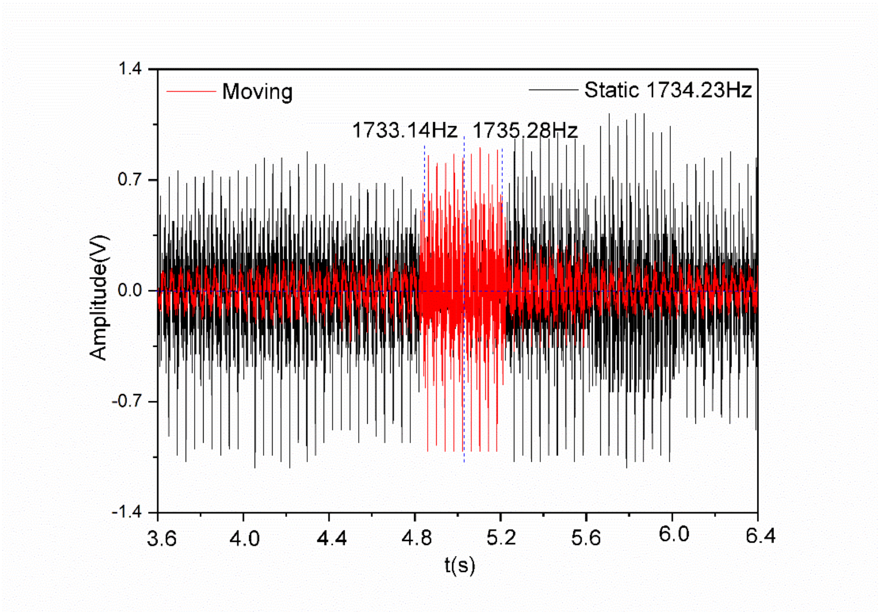

Supplementary Figure S10. The results for pitch 2 at the speed of 0.1 m/s

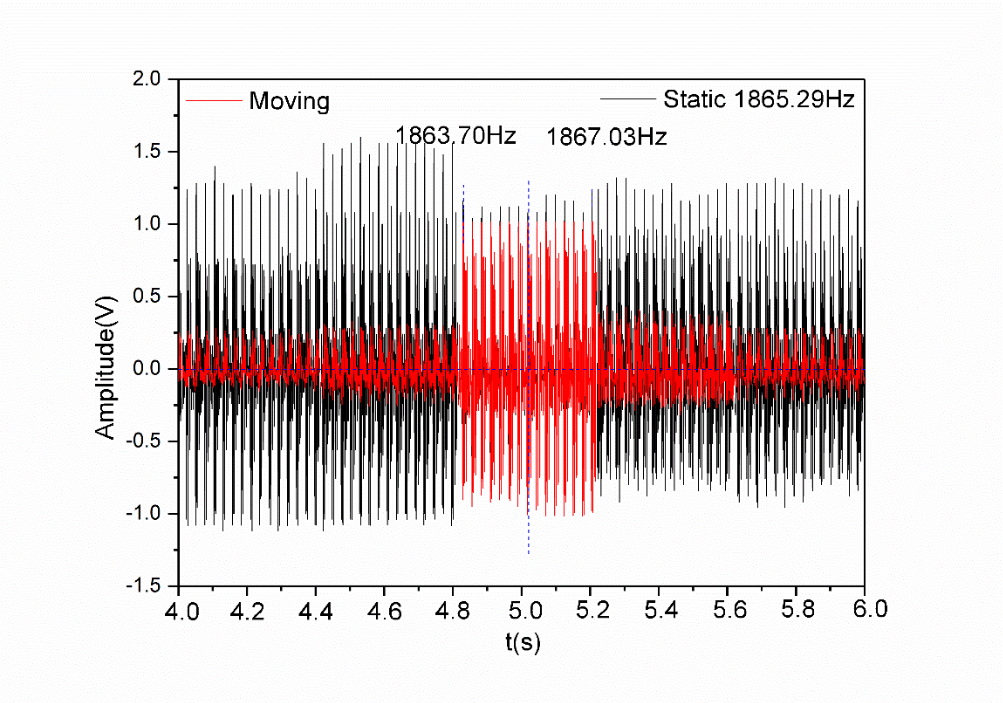

Supplementary Figure S11. The results for pitch 3 at the speed of 0.1 m/s

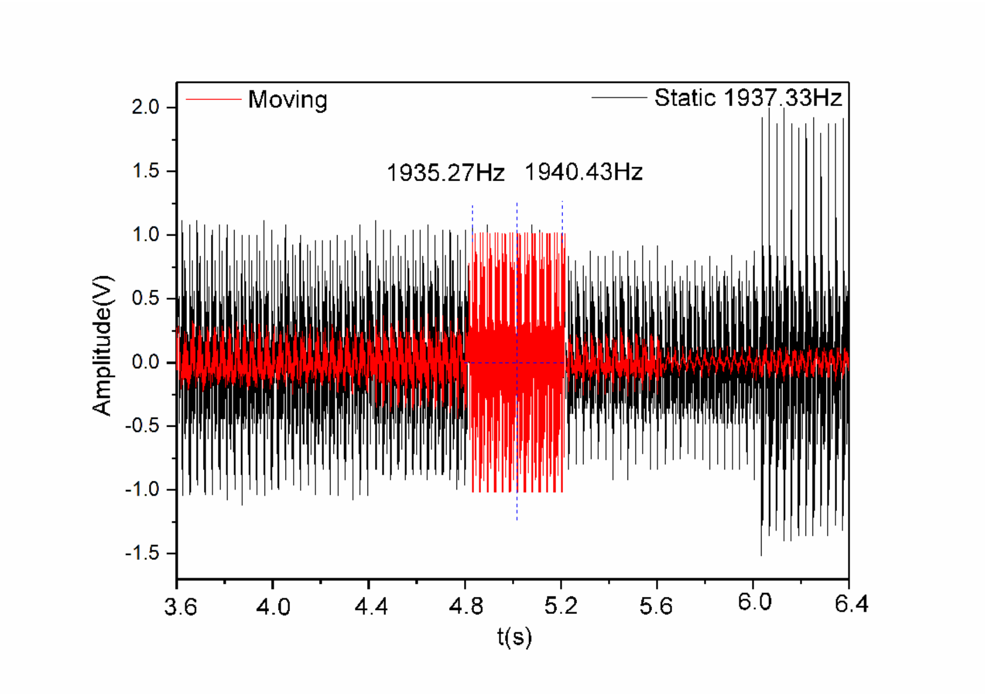

Supplementary Figure S12. The results for pitch 5 at the speed of 0.1 m/s
